# Restoring Faded Writing

**Published:** 1871-04

**Authors:** 


### RESTORING FADED WRITING.

In case a doctor's prescription should become illegible, dip a feather in tincture of galls, or a solution of ferro-cyanide of potassium, slightly acidulated with hydrochloric acid; apply either of these washes as nearly as possible to the ink lines, and thus prevent the spreading of the ink.

